# The contralateral-based submental artery island flap: feasibility and oncological safety in oral cancer–related defect reconstruction

**DOI:** 10.1007/s00784-023-05103-1

**Published:** 2023-06-14

**Authors:** Jingxin Ma, Xuefan Zhai, Min Huang, Peiyao Li, Yujie Liang, Daiqiao Ouyang, Yu-xiong Su, Wei-fa Yang, Guiqing Liao, Sien Zhang

**Affiliations:** 1 Department of Oral and Maxillofacial Surgery, Hospital of Stomatology, Guangdong Provincial Key Laboratory of Stomatology, Guanghua School of Stomatology, Sun Yat-sen University, Guangzhou, China; 2 Division of Oral and Maxillofacial Surgery, Faculty of Dentistry, The University of Hong Kong, Hong Kong Special Administrative Region, China

**Keywords:** Contralateral submental artery island flap, Oral squamous cell carcinoma, Oncological safety, Reconstruction

## Abstract

**Objectives:**

Oncologic risk is a serious concern of submental artery island flaps. Here, we introduce the contralateral-based submental artery island flap (C-SAIF) and demonstrate its feasibility and long-term oncological safety in reconstructing oral cancer–related defects.

**Methods:**

An anatomical study was performed concentrating on the pedicle length in seven cadavers. Then, a retrospective study was carried out on C-SAIF patients operated on by a single team. The standard surgical technique of C-SAIF was conducted. Outcomes including operative time, length of hospital stay, volume of intraoperative blood loss, and scores of the Multidisciplinary Salivary Gland Society (MSGS) questionnaire were compared with a similar cohort reconstructed with anterolateral thigh free flap (ALTF). In addition, oncological outcomes were evaluated by the 5-year cumulative survival rate between C-SAIF and ALTF patients.

**Results:**

The pedicle length of C-SAIF was sufficient for the flap to be extended to the contralateral oral cavity. Fifty-two patients were included in the retrospective study, and nineteen of them underwent reconstruction with C-SAIF. The operative time of C-SAIF was shorter (*p* = 0.003), and the intraoperative blood loss was less (*p* = 0.004) than that of ALTF. There was no difference in MSGS scores. The results of survival analysis revealed comparable survival curves for the two groups in terms of overall survival, disease-specific survival, and disease-free survival.

**Conclusion:**

C-SAIF is a feasible and reliable flap for reconstructing oral cancer–related defects. Moreover, it is an effective island flap to preserve the perforator and pedicle without compromising oncological safety.

**Supplementary Information:**

The online version contains supplementary material available at 10.1007/s00784-023-05103-1.

## Introduction

The oral cavity is the most common anatomic site where squamous cell carcinomas arise within the head and neck [1]. A relatively high frequency of regional lymph node metastasis is the key feature of oral cancer. Tumor resection, regional lymph node dissection, and defect reconstruction are the standard procedures for OSCC surgical treatment [2].

Since the 1980s, free tissue transfer has gradually gained the workhorse position in head and neck reconstruction [3]. Free flaps, such as anterolateral thigh (ALT) flap, radial forearm (RF) flap, and lateral arm flap (LAF), are well-vascularized tissues used to reconstruct oral oncology defects. However, there are several potential risk factors for free flap complications, such as pulmonary comorbidity [4], preoperative radiation [5], and diabetes [6]. In addition, not every patient is suitable for microvascular procedures, especially elderly or poor recipient vascular condition patients. In addition, extended free flap harvesting brings potential complications, including hematoma, wound infection, and hypoesthesia, which might prolong operative time and inpatient hospital stay [7]. Therefore, local pedicle flaps provide an effective and less demanding alternative in oral cancer–related defect reconstruction, for example, submental island flap and nasolabial flap.

In 1993, Martin et al. [8] introduced the submental flap as a pedicled flap with good skin color matching and a wide rotation radian. The submental artery island flap has advantages over the free flap in terms of ease of dissection and fewer complications [9]. Without microvascular anastomosis, it demonstrates a shorter operative time and less intensive postoperative monitoring in past studies [10, 11]. However, OSCC-related reconstruction using submental flaps is a controversial issue due to the proximity to level I cervical lymph nodes. Some researchers concluded that adequate neck dissection can be fulfilled with flap elevation and that SIF reconstruction is not associated with locoregional recurrence [2, 12–14], while some researchers suggested that submental flaps should be carefully indicated in oncological reconstruction, especially in patients with suspicion of lymph node involvement at level I [15, 16]. For oral cancer patients, it is a serious concern that the ipsilateral-based submental artery island flap may conflict with lymph node dissection, thus causing poor prognosis. It would be a disaster if the metastatic lymph node was ruptured and disseminated during pedicle preservation. Advanced skills are required during perforator preservation and lymph node dissection. Is there an easier way?

Considering the difficulties in achieving adequate cervical lymph node dissection while meticulously preserving the pedicle, it would technically be easier and safer to harvest the flap based on the vascular pedicle from the contralateral side. The primary aim of this research is to propose a modification of the flap preparation and to investigate the feasibility and oncological safety of the contralateral-based submental artery island flap (C-SAIF) for the reconstruction of oral cancer–related defects.

## Materials and methods

### Patient cohort

The medical records of patients who underwent surgical resection and soft tissue reconstruction of OSCC-related defects using C-SAIF between December 2014 and May 2021 were extracted. All of the patients were treated at the Department of Oral and Maxillofacial Surgery, Hospital of Stomatology, Sun Yat-sen University, Guangzhou, China. For comparative analyses, a group of patients with ALTF was established. Matching criteria were pathological tumor stage (pT), pathological nodal stage (pN), primary site, age, and sex. The exclusion criteria were as follows: (1) patients with any history of surgical treatment or chemoradiotherapy for the primary lesion; (2) patients with apparent lymph node metastasis at the contralateral neck of the primary lesion; and (3) patients with terminal-staged tumors or large area defects unsuitable for local flap reconstruction. After screening, fifty-two patients with oral squamous cell carcinomas involving the buccal mucosa, tongue, floor of mouth, and mandibular gingiva were involved in this retrospective study. A total of 19 patients were included in the C-SAIF group. Thirty-three patients with primary OSCC were included in the ALTF group.

Patients with pN + and high-grade diseases received adjuvant radiotherapy within 6 weeks after surgery. An uneventful follow-up after treatment was consecutively enrolled. The cutoff date for all follow-up encounters among surviving patients was September 30, 2021.

The evaluations of regional neck lymph node condition were carried out by preoperative imaging examination, and the final results were determined by pathological diagnosis. Tumor staging was based on clinical and imaging findings and pathology results corresponding to the eighth edition of the American Joint Committee on Cancer staging. This study was approved by the institutional Ethics Committee of Hospital of Stomatology, Sun Yat-sen University. The present study followed the guidelines set forth by the Declaration of Helsinki.

### Anatomical study

An anatomical study concentrating mainly on the length of the vascular pedicle was performed to preliminarily investigate the feasibility of C-SAIF in reconstructing defects of the oral cavity, especially in regions demanding longer vascular pedicles, such as the buccal and oropharynx.

Seven formalin-preserved adult cadavers were dissected. Each cadaver was measured three times, and the average value of these measurements was accepted as the final value. The following parameters were measured: (1) length of the submental artery, (2) length of the facial-submental artery, (3) distance between the contralateral mandibular angle and the origin of the facial artery, (4) distance between the contralateral mouth corner and the origin of the facial artery, and (5) diameter of the submental artery at its origin. The length of the submental artery was defined as the distance between the distal end of the submental artery flap perforator and the origin of the submental artery, and the length of the facial-submental artery was defined as the distance between the distal end of the submental artery flap perforator and the origin of the facial artery along the vessel. We measured the length of the artery after ligation of other branches, and the vessel was stretched without tension. The spatial location of the submandibular gland before ligation of the branches and the details of the measurement are available in the supplementary information.

### Surgical technique

**Fig. 1:**
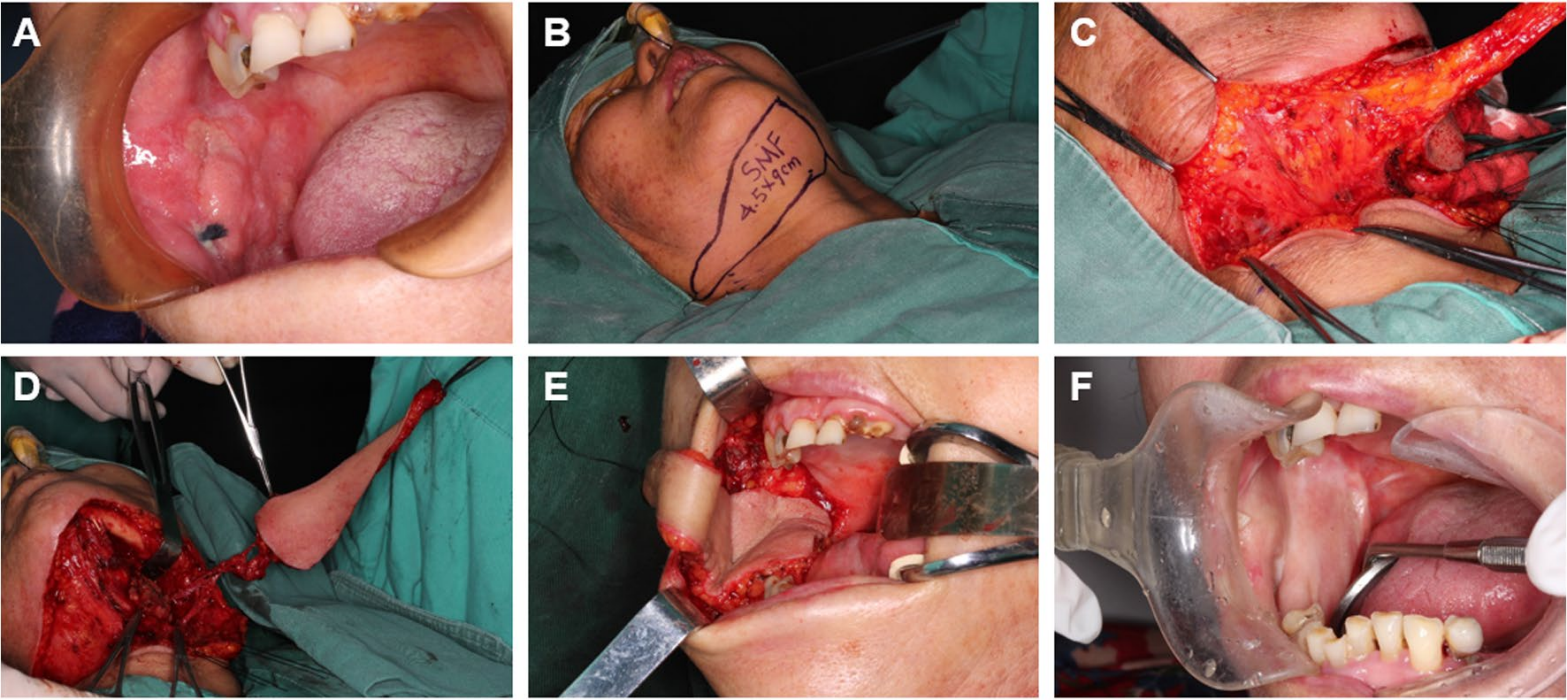
Surgical technique: A typical case of buccal defect reconstruction using a C-SAIF. **A** Primary lesion at the buccal mucosa. **B** The maximum length and the flap in the submental area were outlined in line with the size of the anticipated defect. **C** At the primary lesion side, we raised the flap in the superior layer of the platysma to further ensure that no adjacent lymph nodes were elevated. **D** The flap was elevated after the pedicle along the facial vessels was isolated and dissected. **E** The flap was tunneled and delivered to the defect area. **F** The C-SAIF 32 months after surgery

**Fig. 2:**
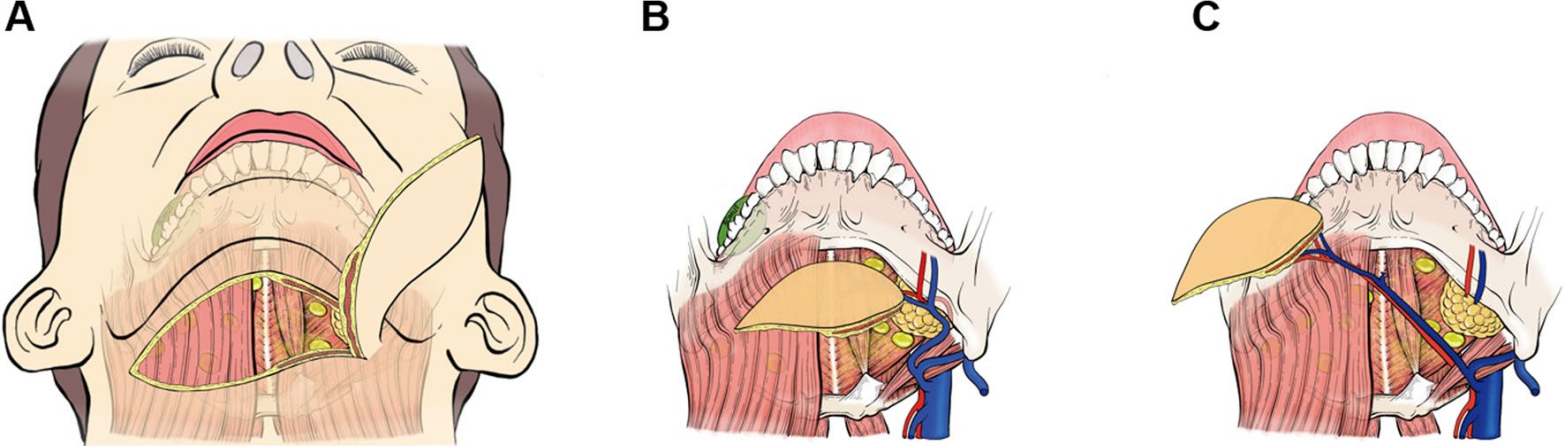
Highlights of surgical techniques raising C-SAIF. **A** The skin paddle was raised upward to the platysma level without disturbing the level I dissection. **B** Oral cancer on the right side was colored green. Perforators and pedicle vessels were identified and dissected on the contralateral (left) side. **C** The distal ends of the facial vessels and glandular branches were ligated to allow free mobility of the perforator island flap. With a long pedicle, the C-SAIF could be extended to the right side

The patient was placed in the central position under general anesthesia. Extended resection of the primary lesion with a safety margin was carried out (Fig. 1A). Tumor-free margins were confirmed by intraoperative frozen sections. After local tumor resection, the flap in the submental area was outlined according to the defect size and the maximum length and width were taken by a pinch test allowing primary closure (Fig. 1B). The highlights of the C-SAIF raising are shown in Fig. 2. A horizontal incision was drawn 2 cm below the inferior margin of the mandible on the contralateral side. The marginal mandibular branch was carefully identified and protected. At the side of the primary lesion, we raised the flap in the superior layer of the platysma to ensure that no adjacent lymph nodes were elevated with the flap (Fig. 1C). The skin paddle was raised upward from the platysma from the contralateral side of the mandibular angle to the anterior belly of the digastric (Fig. 2A). Over the lower border of the mandible, the facial artery and vein were exposed and ligated below the nerve. The anteromedially directed submental artery and vein were identified and dissected away from the submandibular gland. The facial artery branches going into the submandibular gland were ligated to allow C-SAIF, and branches from the lingual artery were protected to preserve the blood supply of the submandibular gland. The facial vein was dissected down to the jugular vein. From the anatomical study, we confirmed some constant perforators in the submandibular triangle, which passed through the angle of the mandible and the contralateral digastric. After the perforator was identified, the skin paddle was raised upward to the platysmal level without disturbing the level IA dissection. The perforator island flap was elevated without the anterior belly of the digastric muscle (Fig. 1D). With a long pedicle, the C-SAIF was tunneled and delivered to the contralateral defect area (Fig. 1E). The intraoral defect was reconstructed by the island flap. More pictures of typical cases are available in the supplementary information.

### Statistical methods

An independent two-sample* t* test was used to compare the mean operative time, hospital stay duration, intraoperative blood loss, and Multidisciplinary Salivary Gland Society (MSGS) scores between the two groups. The Pearson chi-squared test was employed to assess the patient and tumor characteristics. The analyses mentioned above were performed using IBM SPSS advanced statistics (Statistical Package for Social Science), version 23 (SPSS Inc, Chicago, Illinois). The R Project for Statistical Computing was used to calculate survival and determine significance. Kaplan–Meier survival plots and log-rank tests were used to compare the overall survival (OS), disease-free survival (DFS), and disease-specific survival (DSS) between the two groups. The association between patient characteristics and the choice of flap was assessed using inverse probability-weighted (IPW) logistic regressions. IPW survival analyses were conducted to adjust for the effects of tumor stage, nodal stage, age, and sex. Statistical significance was defined as a *p* value < 0.05.

## Results

### Anatomical study of the pedicle length

**Fig. 3:**
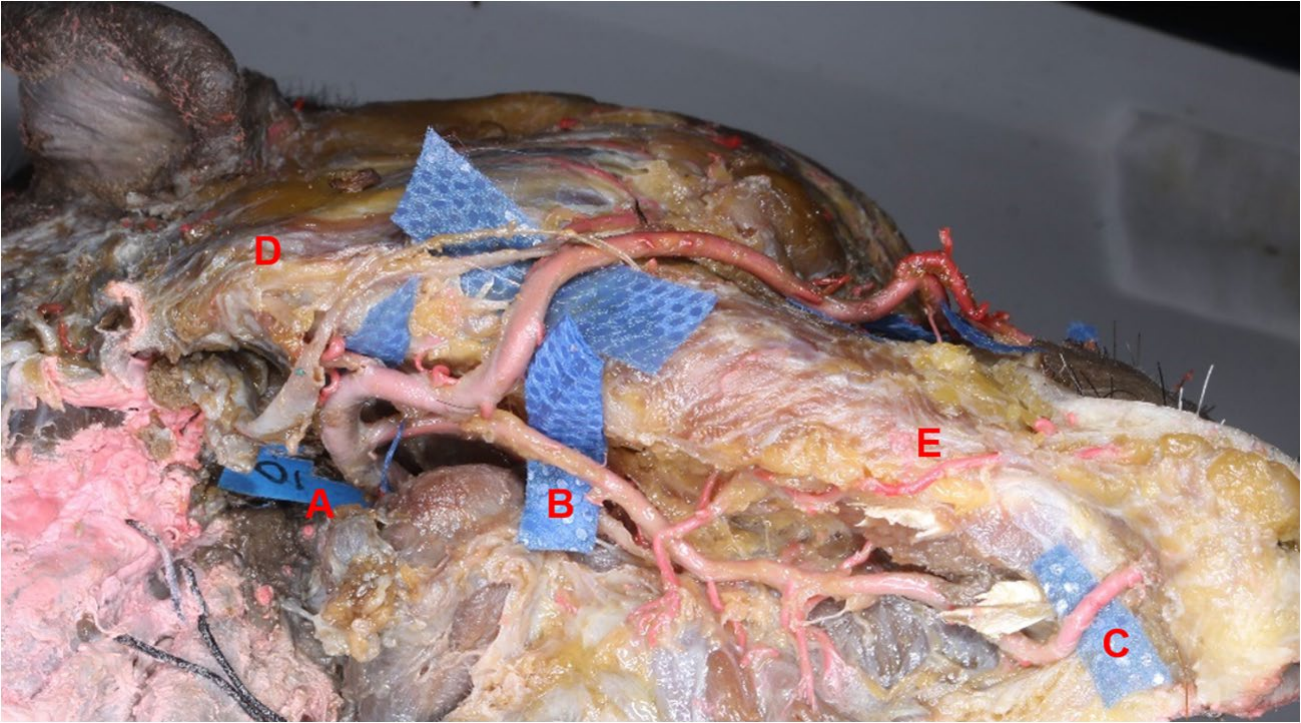
The facial-submental artery. A Facial artery. B Submental artery. C The distal end of the submental artery. D Mandibular angle of the pedicle side. E Perforators of SAIF on the pedicle side

The anatomical results are listed in Table 1. The median length of the facial-submental artery was 102.92 ± 9.53 mm, which was longer than the distance between the mandibular angle and origin of the contralateral facial artery, which was 86.96 ± 7.03 mm. The distance between the contralateral mouth corner and the origin of the facial artery was 99.18 ± 6.18 mm. In addition, the diameter of the submental artery at its origin was 1.49 ± 0.33 mm. Figure 3 shows the trend of the submental artery observed from the bottom.

**Table 1:** Measurements of the facial-submental artery

Description	Minimum (mm)	Maximum (mm)	Median (mm)	Mean ± SD (mm)
Length of the submental artery	53.40	63.44	58.36	58.70 ± 3.99
Length of the facial-submental artery	88.60	112.86	105.35	102.92 ± 9.53
Distance between the contralateral mandibular angle and the origin of facial artery	79.56	99.74	83.21	86.96 ± 7.03
Distance between the contralateral mouth corner and the origin of facial artery	93.46	111.96	96.75	99.18 ± 6.18
Diameter of the submental artery at its origin	1.10	1.55	1.44	1.49 ± 0.33

### Demographics and tumor characteristics

Fifty-two patients were included in the retrospective studies. Nineteen patients underwent reconstruction with C-SAIF, and 33 patients underwent reconstruction with ALTF. A total of 21 women and 31 men were included, with ages ranging from 22 to 84 years and 55 years was the average. All patients underwent resection for squamous cell carcinoma of the oral cavity and received primary reconstruction.

Among all patients, 24 patients were pN0, 28 patients were pN + , and no patients had contralateral neck metastasis, confirmed by postoperative pathology. None of the patients received induction chemotherapy or preoperative definitive radiation therapy. Twenty-eight (53.85%) patients received adjuvant radiotherapy postoperatively. The demographics and tumor characteristics are presented in Table 2.

**Table 2:** Patient and tumor characteristics

Variable	ALTF, *n* = 33 *N* (%)	C-SAIF, *n* = 19 *N* (%)	*p*
Age, years			0.356
≤ 70	29 (87.88)	14 (73.68)	
≥ 70	4 (12.12)	5 (26.32)	
Sex			0.436
Male	21 (63.64)	10 (52.63)	
Female	12 (36.36)	9 (47.37)	
Smoking			0.545
Ever	9 (27.27)	3 (15.79)	
Never	24 (72.73)	16 (84.21)	
Hypertension			0.937
Yes	7 (21.21)	5 (26.32)	
No	26 (78.79)	14 (73.68)	
Primary site			0.424
Buccal	6 (18.18)	3 (15.79)	
Tongue	24 (72.73)	11 (57.89)	
Floor of mouth	2 (6.06)	3 (15.79)	
Mandibular gingiva	1 (3.03)	2 (10.53)	
Tumor stage (pT category)			0.687
T2	20 (60.61)	10 (52.63)	
T3	12 (36.36)	8 (47.37)	
T4a	1 (3.03)	1 (5.26)	
Nodal stage (pNodal category)			0.791
N0	14 (42.42)	10 (52.63)	
N1	6 (18.18)	2 (10.53)	
N2b	13 (39.39)	7 (36.84)	

### Postoperative complications and main outcomes

Two patients with C-SAIF developed wound dehiscence at the recipient site with subsequent complete resolution. Bedside debridement was performed on a C-SAIF, which developed partial necrosis at the most distal extent of the flap. An ALTF required microvascular revision resulting in flap failure, and the defect was filled by a buccal fat pad. Managed with local wound care, the patients mentioned above healed uneventfully.

**Fig. 4:**
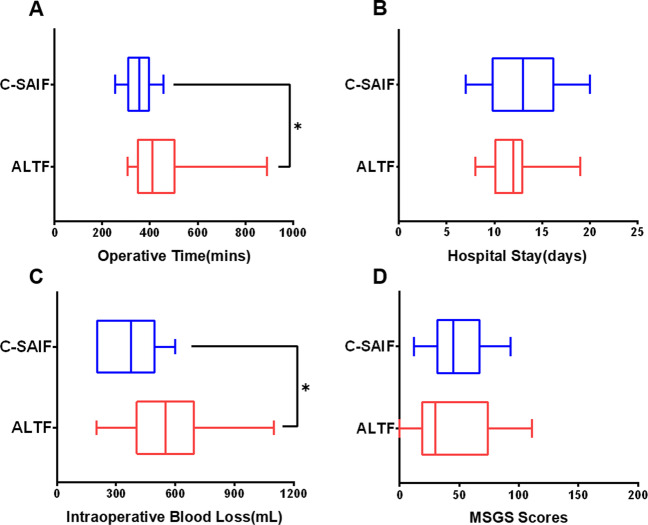
Main outcomes of patients who underwent C-SAIF or ALTF reconstruction. **A** Operative time. **B** Hospital stay. **C** Intraoperative blood loss. **D** MSGS scores

The main outcome variables, including operative time, length of hospital stay, intraoperative blood loss, and MSGS scores, are presented in Fig. 4. The Multidisciplinary Salivary Gland Society (MSGS) is a questionnaire consisting of 20 questions and two systems to quantify symptoms of dry mouth and sialadenitis [17]. We employed the MSGS (0–10 scale version) to evaluate the submandibular gland secretory function of patients who underwent C-SAIF reconstruction and regarded it as a scoring system in which a higher score was more likely related to dry-mouth symptoms. There were 41 questionnaires restored at least 12 months after surgery.

The operative time was shorter at 359 min for C-SAIF than at 448 min for ALTF (*p* = 0.003). The median hospital stay for C-SAIF was 13 days, whereas the observed hospital stay was 12 days for patients who underwent ALTF (*p* = 0.251). The volume of intraoperative blood loss for patients who underwent C-SAIF reconstruction was smaller at 364 mL compared to 546 mL for patients with ALTF (*p* = 0.004). Most patients had relatively low MSGS scores, and there was no difference between groups (*p* = 0.704). The percentage of patients who underwent tracheotomy was 78.95% (15/19) in the C-SAIF group and 87.88% (29/33) in the ALTF group.

### Survival analysis

Survival analyses were conducted for 52 patients, which included 19 and 33 patients in the C-CSAIF and ALTF groups, respectively. In total, there were one case of local recurrence and 2 cases of regional recurrence in the C-SAIF group and 3 local, 2 regional, and 1 distant recurrences in the ALTF group. Approximately 83% of the patients were alive at the time of data collection. No significant difference in the total recurrence was detected (*p* = 1.0). The follow-up time for the patients ranged from 5 to 80 months with a median of 36 months. There were 9 cases of recorded death, with 8 patients dying of the disease and one patient dying of other causes.

**Fig. 5:**
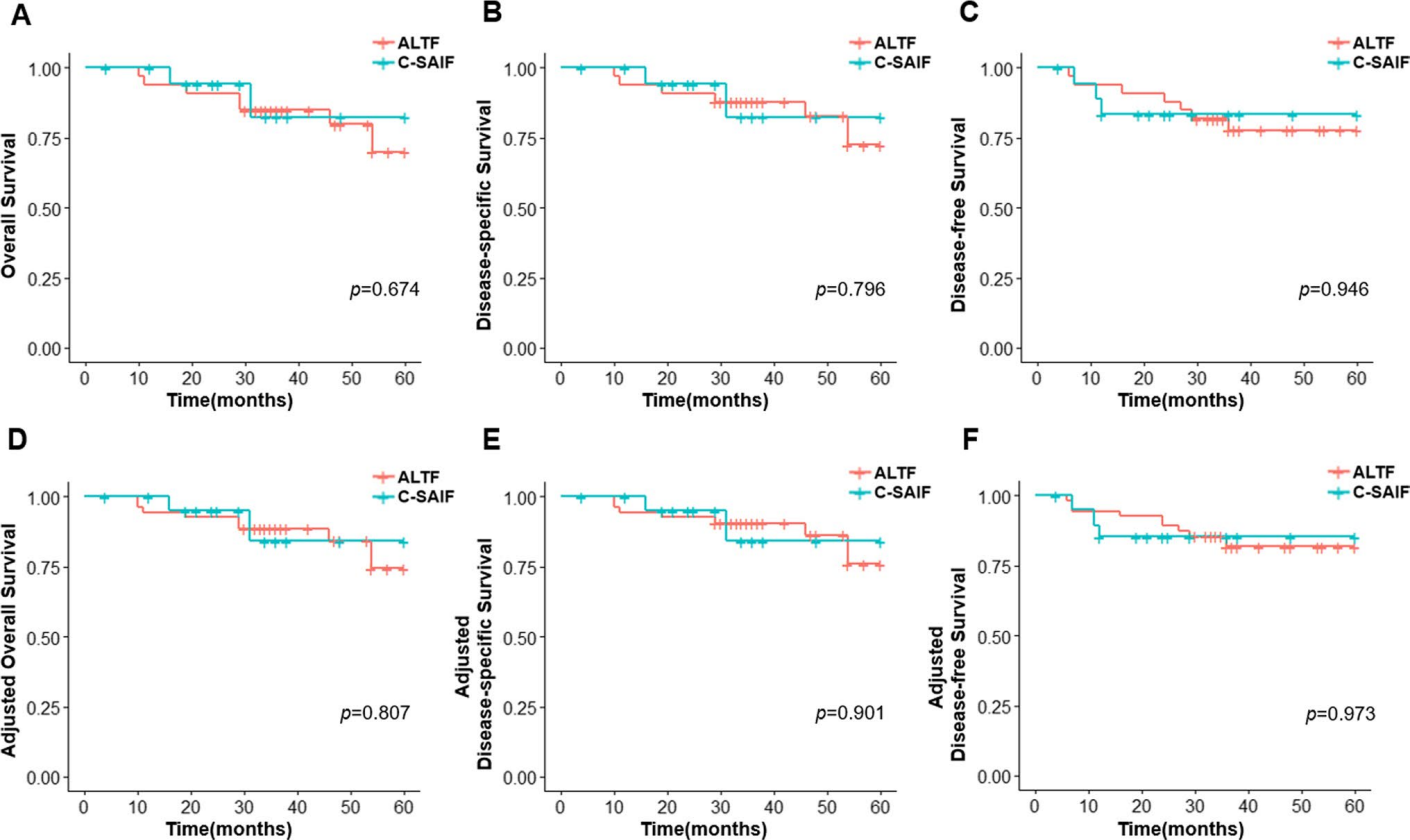
Kaplan–Meier curves of survival analysis of patients treated with C-SAIF or ALTF. **A** Overall survival. **B** Disease-specific survival. **C** Disease-free survival. **D** Adjusted overall survival. **E** Adjusted disease-specific survival. **F** Adjusted disease-free survival. Log-rank tests before and after adjustment in all four survival analyses yielded a *p* value > 0.05

The results of Kaplan–Meier survival plots are shown in Fig. 5. Log-rank tests did not detect any statistically significant difference between the C-SAIF and ALTF groups in terms of OS (*p* = 0.807), DSS (*p* = 0.901), and DFS (*p* = 0.973) after adjustment.

## Discussion

Several reports have shown that SAIF is a reliable flap with good skin color matching and a wide rotation radian [7, 8]. It has become increasingly popular and has been shown to be well suited for defects of the tongue [2, 16, 18], buccal mucosa [2, 16], and lower and mid face [19]. However, concerns about its oncological safety remain. The local nodal basins adjacent to the pedicle bring the possibility of transferring occult cervical lymph node metastases to the recipient site, especially for basic-level surgeons. Additionally, metastatic lymph nodes may be ruptured and disseminated during pedicle and perforator preservation. Here, we introduce an easier and safer method, the contralateral-based submental artery island flap, to prevent this disaster. Our study shows that the pedicle length of the C-SAIF is feasible for reconstructing contralateral oral defects, and flap raising does not disturb ipsilateral neck dissection without compromising oncological safety and long-term prognosis.

Oncological safety is the core concern of SAIF for the reconstruction of cancer-related defects. Shen et al. [20] reported several recurrence cases with metastatic lymph nodes under the skin paddle after SAIF reconstruction. Cariati et al. [16] found that 4 of 9 patients experienced local or cervical relapse in their study. Notably, some surgeons might convert ipsilateral-based SAIF to free flaps when they find some suspected lymph nodes during surgery. It is particularly adventurous for patients with aggressive oral cancer or a high propensity for neck metastases. On the other hand, some researchers suggested that oncological outcomes are not compromised in clinically node-negative oral cancer patients, even in node-positive patients. Wang et al. [2] analyzed the oncological results of pN0 and pN + patients with SAIF reconstruction and concluded that SAIF did not increase the risk of recurrence in patients with T1-2 OSCC with careful neck dissection and appropriate postoperative adjuvant treatment. However, advanced skills are required during perforator preservation and lymph node dissection. In summary, the concern for oncological safety became the main disadvantage and restricted the scope and indications of SAIF.

Considering the difficulties in achieving adequate cervical lymph node dissection while preserving the pedicle, it would be safer to harvest the flap based on the pedicle from the opposite side. Furthermore, it is easier to spread among surgeons with different experience levels. In our previous studies, the contralateral nasolabial island flap was employed for tongue and buccal reconstruction and emphasized its oncological safety [21, 22]. However, the sacrifice of esthetics and the potential risk of facial nerve injury, especially the marginal mandibular and buccal branches, limit the use of the flap [21]. Thus, as an improvement of local flaps, the contralateral submental artery island flap extends the indications and keeps the advantages of oncological safety, overcoming these short limits.

In reviewing the literature, there are few reports about the use of the C-SAIF. Schonauer et al. [23] described the use of contralateral submental flaps in 4 cases. Xie et al. [24] evaluated the defect area, flap size, and complications of a series of cases with intraoral cancer defects reconstructed with C-SAIF. The oncological outcome of C-SAIF was described first in Amin et al.’s study [25]. However, these studies included a small number of patients or had limited follow-up times, and there was no contrastive survival analysis between SAIF and free flaps. To the best of our knowledge, our work is the first study confirming the long-term oncological safety of C-SAIF by investigating the survival curves and comparing them with free flaps. In terms of OS, DSS, and DFS, there was no significant difference between the C-SAIF or ALT flap groups. Our results revealed that C-SAIF can provide ideal oncological safety for the reconstruction of OSCC-related soft tissue defects.

In terms of the anatomical study, the mean diameter of the submental artery at its origin in the cadavers in our study was close to the value (1.7 mm) reported by Magden et al. [26]. However, to our knowledge, no research has systematically investigated the maximum pedicle length of SAIF. Our study shows that the pedicle length is longer than the distance between the mandible angle and the origin of the contralateral facial artery. This is the first anatomical study to confirm the feasibility of C-SAIF in oral cancer–related reconstruction. Initially, we evaluated the feasibility of C-SAIF. Henceforth, nineteen oral cancer patients were reconstructed by C-SAIF in our clinical institution. There were three cases of buccal defects reconstructed with C-SAIF resulting in a favorable prognosis with no complications, such as limitation of mouth opening or obvious deviation of mouth angle. Our anatomical and clinical study indicates that C-SAIF is feasible for intraoral defect reconstruction.

In this study, C-SAIF was found to be associated with a shorter operative time and less intraoperative blood loss than ALT free flap. The average operative time was 359 min compared to 448 min for ALTF in our group. Similarly, previous studies reported shorter operative times for SAIF than for free flap transfer [7, 27, 28]. Intraoperative blood loss for patients undergoing C-SAIF reconstruction was remarkably less compared to ALTF, and it was comparable with that of submental artery island flap in a previous study [28]. The MSGS questionnaire did not detect any difference between two groups, indicating that C-SAIF is not associated with xerostomia by preserving the submandibular gland.

One unanticipated finding was that there was no difference in the length of hospital stay or rate of tracheotomy between the groups. As a specialized hospital, there are few shortcomings that are unable to be overcome currently, for example, insufficiency of intensive care units. As a result, we have to select extremely safe airway management to ensure the perioperative safety of patients to the greatest extent. In addition, most patients at our institution underwent preoperative examination after admission to the hospital. The reasons mentioned above contribute to the fact that the length of hospital stay was longer than most reported cases (8.0–15.9 days) [7, 10, 27]. The advancement of tiered medical services and more precise treatment planning could be helpful to shorten hospitalization. Although the percentage of patients undergoing tracheotomy was not different between the submental flap and free flap groups in a previous study [7], there was a significant decline in the rate of tracheotomy use over their study period.

In this study, we retrospectively evaluated the outcomes of patients undergoing squamous cell carcinoma–related defects of the oral cavity reconstructed concurrently with the C-SAIF to a similar cohort undergoing ALT free flap transfer reconstruction by using a single-institution database. The results showed several advantages of C-SAIF over ALT free flap; namely, this technique independent of microvascular anastomosis was associated with a shorter operative time and with less intraoperative bleeding and resulted in comparable survival to free tissue transfer. These findings suggest that this kind of flap can be used by surgeons of different levels of experience without concerns about the technical difficulties in adequate lymph node dissection while preserving the pedicle. A prospective, randomized study with a sufficient sample size based on multi-institution databases is necessary to evaluate the oncological safety and reliability of contralateral-based submental artery island flaps for the reconstruction of oral cancer–related defects.

## Conclusion

C-SAIF is a feasible and reliable flap for the reconstruction of oral cancer–related defects. It is an effective alternative pattern to preserve the perforator and pedicle without compromising oncological safety.

## Supplementary Information

Below is the link to the electronic supplementary material.

Supplementary file1 (DOCX 2335 KB)

## Data Availability

The data used to support the findings of this study are available from the corresponding authors upon request.
