# Supplementary material for: The contralateral-based submental artery island flap: feasibility and oncological safety in oral cancer–related defect reconstruction
Source: Clin Oral Investig. 2023 Jun 14;27(8):4747–55. doi: 10.1007/s00784-023-05103-1 (PMC10415524; doi:10.1007/s00784-023-05103-1)
Supplement: Supplementary file 1 — Supplementary file1 (DOCX 2335 KB) [file 784_2023_5103_MOESM1_ESM.docx]

**Supplementary Information**

**
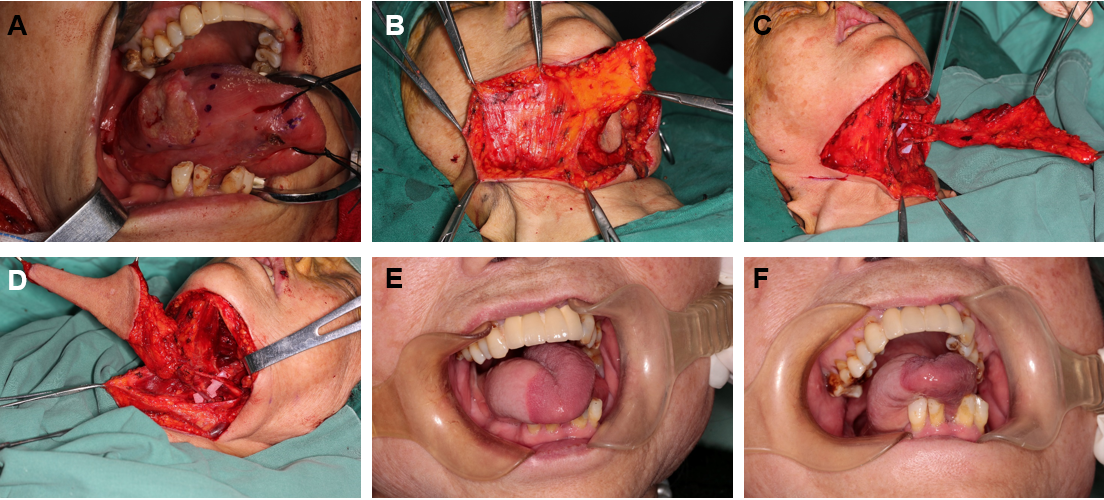
**

Fig.S 1 Typical case of tongue defect reconstruction using an C-SAIF. A. The primary lesion at the buccal mucosa; B. Raise the flap in the superior layer of platysma at the side of primary lesion to further ensure no adjacent lymph nodes being elevated with the flap; C-D. The flap was elevated after the pedicle along the facial vessels were isolated and dissected; E-F. The C-SAIF at the right side of tongue 12 months later.


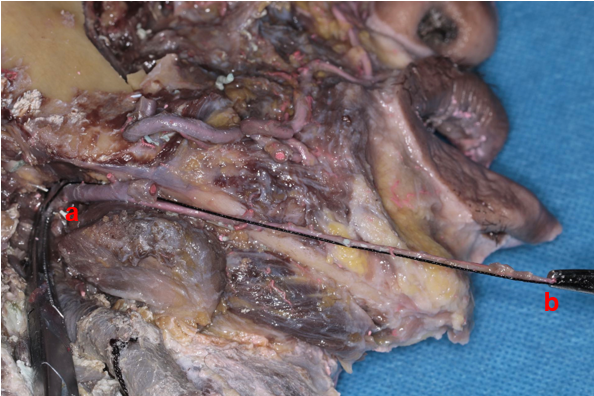


Fig.S 2 We measured the length of the artery from the origin of facial artery to the distal end of submental artery, which represents the theoretical maximum pedicle length of C-SAIF. The length was measured after the vessel had been stretched without tension (an adhered surgical suture as indication). (a)The origin of the facial artery; (b)The distal end of the submental artery.


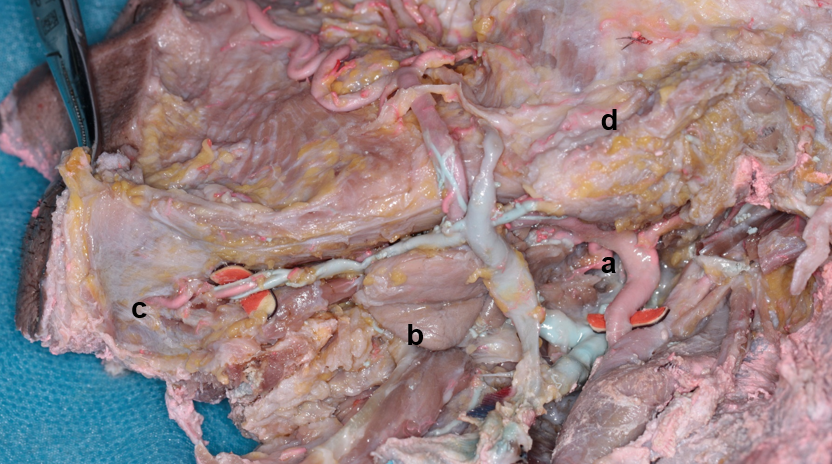


Fig.S 3 The submental artery and submandibular gland. (a)The origin of facial artery; (b)The submandibular gland; (c)The distal end of submental artery;(d) Mandibular angle.
